# Phasing tiny crystals

**DOI:** 10.1107/S2052252513034507

**Published:** 2013-12-31

**Authors:** Jianwei Miao, Jose A. Rodriguez

**Affiliations:** a Department of Physics and Astronomy, and California NanoSystems Institute, University of California, Los Angeles, CA 90095, USA; b Department of Biological Chemistry, UCLA-DOE Institute for Genomics and Proteomics, University of California, Los Angeles, CA 90095, USA

**Keywords:** oversampling, coherent diffraction imaging, X-ray free electron lasers, phase retrieval

## Abstract

For tiny crystals, their diffraction intensities at and between the Bragg peaks become measurable due to the limited number of the unit cells, which can in principle be used to directly phase the crystal structures.

The discovery and interpretation of X-ray diffraction from crystals by von Laue, Henry and Lawrence Bragg about a century ago marked the beginning of an era for visualizing the three-dimensional atomic structure in crystals. Indeed, X-ray crystallography has been fundamental to the development of many scientific fields. However, when an X-ray wave is scattered by a crystal, a detector can measure the magnitude of the scattered wave, but the phase information is lost. This constitutes the well known ‘phase problem’. Over the years, a number of phasing methods have been developed to solve crystal structures. In the January issue of **IUCrJ**, Liu *et al.* (2014 ▶) propose an *ab initio* phasing method to determine the structure of tiny (*i.e.* nanoscale) crystals with incomplete unit cells.

The origin of the proposed method can be traced back to 1952 when Sayre suggested in his half-page paper that measuring the diffraction intensity of a crystal at Bragg peaks as well as between them would suffice to provide the phase information (Sayre, 1952 ▶). However, this idea was virtually ignored by the main stream X-ray diffraction community for nearly a half a century until the first recording and reconstruction of the diffraction pattern of a non-crystalline specimen by Miao and collaborators (1999 ▶), in which the phase problem was solved through a combination of the oversampling method (Miao *et al.*, 1998 ▶) and iterative algorithms (Fienup, 1982 ▶). This experiment created a flurry of research activities, extending the methodology of X-ray crystallography to allow structural determination of non-crystalline specimens and nanocrystals (Robinson & Harder, 2009 ▶; Chapman & Nugent, 2010 ▶; Miao *et al.*, 2012 ▶). The method, known as coherent diffraction imaging (CDI) or lensless imaging, has also become one of the major justifications for the construction of X-ray free electron lasers (XFELs) worldwide (Emma, 2010 ▶; Ishikawa, 2012 ▶). Compared with synchrotron radiation, XFELs have three unique properties: (i) full transverse coherence, (ii) high peak intensity (~10^12^ photons/pulse), and (iii) ultra-short pulse duration (a few to hundreds of femtoseconds), which make it possible to collect the diffraction pattern of individual biological specimens such as viruses and tiny protein crystals before they are destroyed by single XFEL pulses (Neutze *et al.*, 2000 ▶; Chapman *et al.*, 2011 ▶). For tiny crystals, the diffraction intensity at and between the Bragg peaks becomes measurable due to the limited number of the unit cells, which can be used to phase the crystals. The principle of this phasing method was first studied through numerical simulations by Miao & Sayre (2000 ▶), and the first experimental demonstration of phasing an inorganic nanocrystal at low resolution was performed by Robinson and collaborators (2001 ▶).

Spence and collaborators have now applied this oversampling method to *ab initio* phasing of nanocrystals with incomplete unit cells using numerical simulations (Liu *et al.*, 2013 ▶). For X-ray diffraction of a nanocrystal, two main terms other than its lattice contribute to its diffraction pattern (Fig. 1 ▶): the shape transform (*i.e.* the Fourier transform of the crystal shape) and the molecular transform (*i.e.* the Fourier transform of the molecules inside a unit cell). If the intensities at and between Bragg peaks are measured and the number of the unit cells of the nanocrystal is estimated, the shape transform can be divided out from the recorded diffraction pattern to recover the molecular transform, which can then be directly phased to obtain the molecular structure using the oversampling method with iterative algorithms. However, applying this method to tiny protein crystals with XFELs faces two challenges. First, for many protein nanocrystals, there may exist incomplete unit cells at the surface layers of the crystals. Second, when large numbers of nanocrystals are used in an experiment, the variations in crystal size and shape cannot be avoided. These two factors can, in principle, make it very difficult to extract the molecular transform accurately from the recorded diffraction pattern. Using numerical simulations, Liu *et al.* (2013 ▶) have now found that, by merging a large number of nanocrystals, the incomplete unit cells and the crystal size variations do not prevent the oversampling method from recovering the molecular transform with the assumption that certain unit-cell types are preferred (Fig. 1 ▶). This solution to the phase problem is advantageous in that it does not require atomic resolution data, chemical modification of samples, or modulation of X-ray energies during data collection. Of most interest to crystallographers is the potential for such a method to work on even the smallest crystals with XFELs. However, the performance of this approach is indelibly linked to the quality of the recorded intensities between Bragg peaks. This stringent requirement is strained by the intrinsic properties of the intensity between Bragg peaks; it rapidly decays even at small distances away from Bragg peaks. Therefore, the next big challenge is to demonstrate this *ab initio* phasing method on experimental data obtained from the tiniest of protein crystals collected from XFELs.

Over the past century, X-ray crystallography has made a tremendous impact in physics, biology, chemistry, mineralogy, geosciences and life sciences. The last several decades have also witnessed three important developments related to X-ray crystallography. First, advanced X-ray sources, such as synchrotron radiation, XFELs, diffraction-limited storage rings and tabletop high harmonic generation (HHG) (Emma, 2010 ▶; Ishikawa, 2012 ▶; Popmintchev *et al.*, 2012 ▶), have been under rapid development worldwide. Second, the methodology of X-ray crystallography has been extended to allow structural determination of non-crystalline specimens and nanocrystals (Miao *et al.*, 1999 ▶; Robinson *et al.*, 2001 ▶). Third, high dynamic range detectors with single photon sensitivity have been developed and computational power has been dramatically increased. While many issues related to these developments remain to be solved and new ideas are also needed, it is safe to predict that X-ray crystallography in the next century will likely be at least as exciting as in the past one.

## Figures and Tables

**Figure 1:**
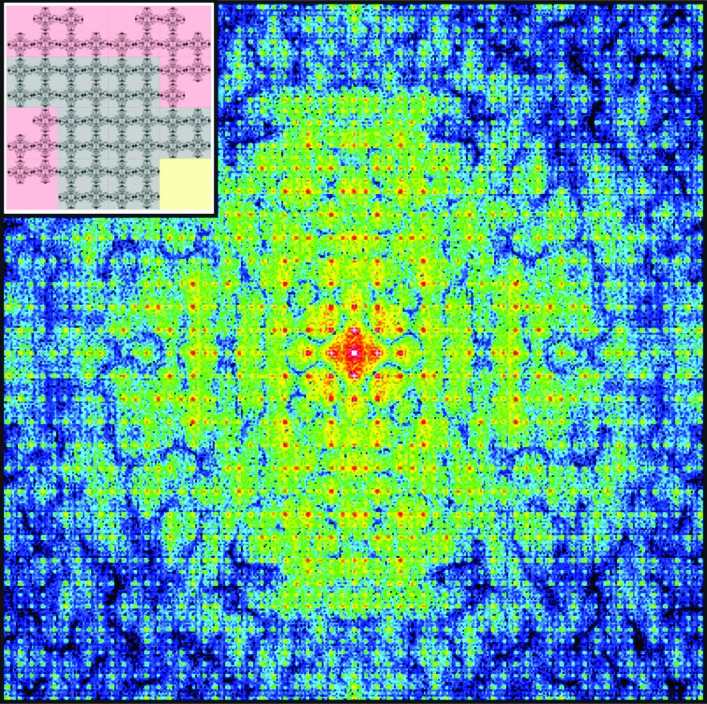
A simulated X-ray diffraction pattern of a two-dimensional nanocrystal, containing a 4 × 4 unit-cell lattice of a hydrophobin molecule (PDB ID 2gvm), in which the underlying molecular transform is visible, delineated by the intensity between the Bragg peaks. An inset shows the structure of the two-dimensional lattice with outer cells labeled as partially occupied (red), unoccupied (yellow) and fully occupied (gray).
